# Prognostic Value of a New Integrated Parameter—Both Collateral Circulation and Permeability Surface—in Hemorrhagic Transformation of Middle Cerebral Artery Occlusion Acute Ischemic Stroke: Retrospective Cohort Study

**DOI:** 10.3389/fnagi.2021.703734

**Published:** 2021-08-25

**Authors:** Chanchan Li, Xiaozhu Hao, Luyi Lin, Chengfeng Sun, Hai Yu, Zhenwei Yao, Xiaoyuan Feng, Yanmei Yang

**Affiliations:** ^1^Department of Radiology, Huashan Hospital, Fudan University, Shanghai, China; ^2^Department of Neurology, Huashan Hospital, Fudan University, Shanghai, China

**Keywords:** collateral circulation, stroke, angiography, perfusion imaging, cerebral hemorrhage

## Abstract

**Background:**

Multimodal CT, including CT angiography (CTA) and CT perfusion (CTP), was increasingly used in stroke triage. This study was to determine the relationship between a new integrated parameter—both collateral circulation and relative permeability surface (PS)—and the hemorrhagic transformation (HT) in acute ischemic stroke (AIS) with middle cerebral artery occlusion (MCAO).

**Methods:**

We retrospectively reviewed consecutive AIS patients with MCAO who underwent baseline CTA/CTP within 4 h of symptom onset and follow-up susceptibility-weighted imaging (SWI) within 3 weeks. Collateral circulation was assessed on the baseline CTA. Baseline CTP data were postprocessed to generate PS parameter. The patients with poor collateral circulation and at the same time with high relative PS were classified as the group of both poor collateral circulation and high relative PS. HT was defined according to European Cooperative Acute Stroke Study II criteria on follow-up SWI imaging. Multivariate logistic regression analysis was performed using HT as an outcome variable.

**Results:**

The group of patients with both poor collateral circulation and high relative PS was thirteen and thirty-three (52%) developed HT of the final cohort sixty-three AIS patients with MCAO. Multivariate logistic analysis revealed the new integrated parameter—both collateral circulation and relative PS (odds ratio, 16.59; 95% confidence interval, 13.09–19.10; *P* < 0.001) was independent predictor of HT. The area under the curve was 0.85 (95% confidence interval, 0.81–0.89). The sensitivity was 57%, specificity 97% and positive predictive value 92%, negative predictive value 58%.

**Conclusions:**

For AIS patients with MCAO, these with poor collateral circulation on CTA and at the same time with high relative PS on CTP were at high risk for HT.

## Introduction

Hemorrhagic transformation (HT) is an unwanted complication of acute ischemic stroke (AIS) that may severely worsen the prognosis (Knight et al., 2019). Multimodal CT, including CT angiography (CTA) and CT perfusion (CTP), is a part of routine acute stroke care in many stroke centers. Numerous studies have tried to determine clinical and imaging parameters associated with HT so as to identify patients at highest risk for thrombolytic or endovascular therapies. Indeed, risks factors for HT have been investigated and included higher age, higher stroke severity, large vessel occlusion and collateral score on CTA and permeability surface (PS) on CTP et al. (Hom et al., 2011; Bivard et al., 2016; Marsh et al., 2016; Wang et al., 2016; Kalinin et al., 2017; Li Q. et al., 2017; Li Y. et al., 2017; Liu et al., 2017; Puig et al., 2017). However, many studies taken it for granted yet could not confirm the cause-and-effect relationship between collateral circulation and PS for these two parameters were simultaneously acquired (Li Q. et al., 2017; Puig et al., 2017; Horsch et al., 2018). Therefore, it was inappropriate to draw a conclusion that inadequate collateral circulation contributed to the increased PS or to identify collateral circulation as a confounding factor in the HT prediction model.

In this study, we integrated the collateral circulation and PS into a new parameter—both collateral circulation and relative PS. We hypothesized that this new parameter contributes to HT and we aimed to determine the relationship between this new parameter and HT in AIS with middle cerebral artery occlusion (MCAO).

## Materials and Methods

### Study Population and Design

A single-center retrospective cohort study was conducted in consecutive AIS patients, according to Guidelines for Diagnosis and Treatment of Acute Ischemic Stroke in China 2014, presenting to the Stroke Unit of Huashan Hospital, Fudan University between January 2016 and December 2019. Patients were included if the following inclusion criteria were met: (1) age > 18 years and < 85 years; (2) National Institutes of Health Stroke Scale (NIHSS) was 4 to 22 at admission; (3) time from symptom onset to no-contrast CT (NCCT)/CTP/CTA was within 4 h and before recanalization treatment; (4) AIS with middle cerebral artery (MCA)-M1 occlusion documented on CTA; (5) the admission NCCT showed no evidence of intracerebral hemorrhage; (6) time from symptom onset to follow-up susceptibility-weighted imaging (SWI) was within 3 weeks. Patients with one or more of the following conditions were excluded: (1) history of brain tumor or brain surgery; (2) ischemic stroke in the bilateral cerebral hemispheres; (3) low imaging quality; (4) abnormal endovascular procedure including inappropriate thrombectomy and impaling vessels.

This study was approved by the Ethics Committee of Huashan Hospital, Fudan University (2020-939) and was registered at the Chinese Clinical Trial Registry (ChiCTR2000035575). Informed consent was waived for collection and analysis of preexisting data. This study was carried out in accordance with the Declaration of Helsinki.

### Clinical Variables

The following clinical variables were collected for each patient: sex, age, admission NIHSS, risk factors (hypertension, hyperlipidemia, diabetes mellitus, atrial fibrillation, previous stroke, and current smoking), recanalization therapy (intravenous thrombolysis and endovascular thrombectomy) and time to recanalization therapy, time to imaging.

Patients without contraindication to thrombolysis received intravenous recombinant tissue plasminogen activator (rt-PA) treatment. Eligible patients without extensive early ischemic signs on pre-interventional scans received endovascular treatment. Patients with contraindications to thrombolysis received supportive care.

### Image Acquisition

All patients underwent baseline NCCT/CTP/CTA within 4 h of symptom onset and follow-up SWI within 3 weeks. Whole-brain NCCT, CTP, and CTA were simultaneously performed on a 256-section CT scanner (Brilliance iCT, Phillips Medical Systems, Cleveland, OH, United States) as follows: Jog mode, 120 kVp, 150 mAs, field of view 220 mm, matrix 512 × 512. A dual-head power injector (Stellate Injection System, Indianola, PA, United States) was used to inject 40 ml of non-ionic contrast medium (Ultravist, iodine 370 mg I/ml; Bayer Healthcare, Berlin, Germany) at 5 ml/s followed by 20 ml saline with 5-s (CTP) and 8-s (CTA) delay. CTP included a 50-s scan reconstructed at 0.4-s intervals to produce a series of 312 sequential images for 13 sections, covering a total of 120 mm from the foramen magnum to the lateral ventricles. SWI was performed on a 3.0T superconducting magnetic resonance imaging (MRI) scanner (Verio, Siemens AG, Erlangen, Germany).

### Image Analysis

**FIGURE 1:**
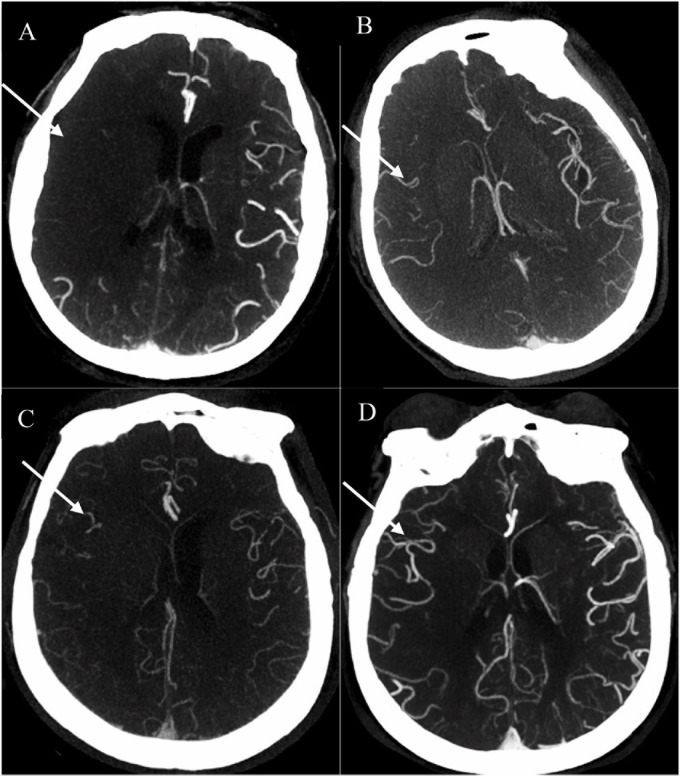
Examples of collateral circulation from 0 to 3 (white arrow). **(A)** 0 = absent collateral, **(B)** 1 = collateral filling < 50% of the occluded territory, **(C)** 2 = collateral filling > 50% < 100% of the occluded territory, and **(D)** 3 = collateral filling 100% of the occluded territory. All four patients were acute ischemic stroke (AIS) with right middle cerebral artery (MCA)-M1 occlusion. AIS, acute ischemic stroke; MCA, middle cerebral artery.

Baseline CTA data were processed using the Phillips Brilliance Workspace portal software (Vision 5.0.2), including 10-mm axial and multi-planar maximum intensity projection reconstructions. The leptomeningeal collateral circulation was graded according to a previously reported scoring system on a scale from 0 to 3: 0 = absent collateral, 1 = collateral filling < 50% of the occluded territory, 2 = collateral filling > 50% < 100% of the occluded territory, and 3 = collateral filling 100% of the occluded territory (Figure 1). The results were dichotomized into poor collateral circulation (a score of 0 or 1) versus good collateral circulation (a score of 2 or 3) (Tan et al., 2009). MCA-M1 occlusion was defined as a main MCA trunk occlusion before the bifurcation, with or without ipsilateral internal carotid artery occlusion.

**FIGURE 2:**
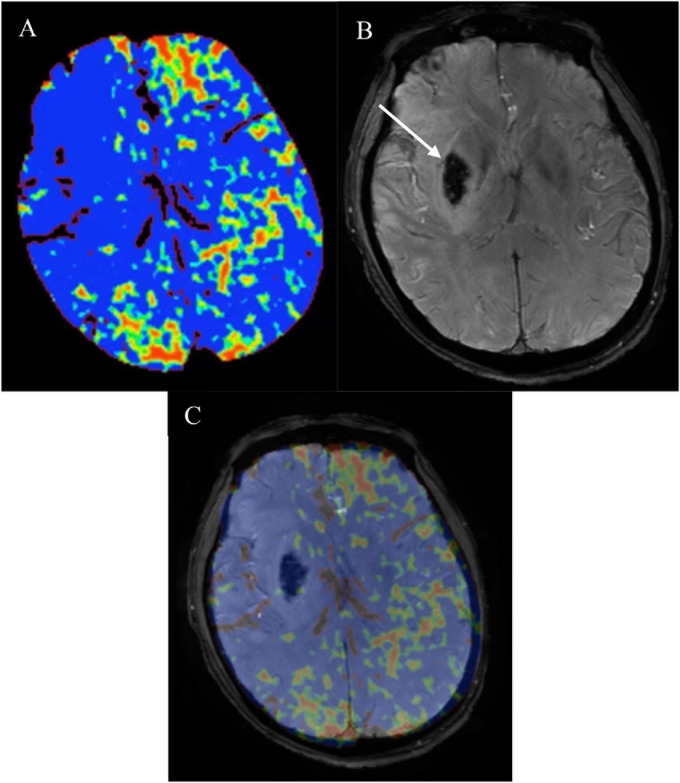
**(A)** CT perfusion permeability surface (PS) map, follow-up **(B)** susceptibility-weighted imaging (SWI) in the same patient as in Figure 1B and **(C)** co-registered map. Obviously increased **(A)** PS on the affected side compared with the contralateral normal side. Follow-up **(B)** SWI showed hemorrhagic transformation (HT) (white arrow) which belonged to parenchymal hematoma (PH) 2. PS map and follow-up SWI image were co-registered automatically using OpenCV 2.4.9. Then the regions of interest (ROIs) were drawn within the HT region and the corresponding contralateral region. Relative PS were calculated by normalizing the values in the HT region with those in the contralateral mirror region. PS, permeability surface; SWI, susceptibility-weighted imaging; HT, hemorrhagic transformation; PH, parenchymal hematoma; ROIs, regions of interest.

Postprocessing of baseline CTP source images was performed using Phillips brain perfusion software (Vision 5.0.2) to generate parametric maps of PS (Figure 2A). The adiabatic approximation tissue homogeneity model and a deconvolution technique were used to calculate CTP parameters. HT was defined as a hypointense susceptibility effect-induced area in the ischemic region on follow-up SWI (within 3 weeks) (Figure 2B). If present, HT was classified according to the European Cooperative Acute Stroke Study II classification into four subtypes, that is, hemorrhagic infarction (HI) 1 and 2 and parenchymal hematoma (PH)1 and 2. Symptomatic intracerebral hemorrhage (SICH) was identified as PH with worsening of neurological deficit ≥ 4 points on the NIHSS that was temporally related to its imaging appearance (Larrue et al., 2001). Baseline PS maps and follow-up SWI images were co-registered automatically using OpenCV 2.4.9 (Figure 2C). Then the regions of interest (ROIs) were drawn within the HT region and the corresponding contralateral region. This method made the ROI on the PS maps on the affected side were in the exact location where the HT happened. Relative PS were calculated by normalizing the values in the HT region with those in the contralateral mirror region. The results were dichotomized into high relative PS (≥2.89) versus low relative PS (< 2.89) (Li Q. et al., 2017).

Two radiologists (CL and XH) who were blinded to the study groups independently assessed the MCA-M1 status and collateral circulation. Two radiologists (LL and CS) assessed the follow-up SWI for HT and relative PS in selected ROIs. According to the new parameter—both collateral circulation and relative PS, the patients with poor collateral circulation and at the same time with high relative PS were classified as the group of both poor collateral circulation and high relative PS.

### Statistical Analysis

Statistical analyses were performed using SPSS 19.0 software (SPSS, Chicago, IL, United States). Quantitative variable was expressed as mean ± standard deviation and categorical variable was expressed as proportion. Differences were assessed using the χ2 test (categorical variables), Student’s-*t* test (variables with normal distribution), or Mann–Whitney rank test (variables without a normal distribution). Univariate and multivariate logistic regression analysis were performed using HT as an outcome variable. Because we had only a limited number of outcomes and only three variables could be selected maximally to have at least 10 outcomes per variable. Variables analyzed were age, admission NIHSS, the new parameter—both collateral circulation and relative PS (Horsch et al., 2018). Interrater reliability was evaluated using weighted *k* values for ordered categorical variables. All *P* < 0.05 was considered significant.

## Results

Sixty-three patients were included in this study from January 2016 to December 2019. Study flow chart was shown in Supplementary Figure I. The baseline characteristics had no significant difference between the patients involved and not involved in this study (Supplementary Table I). Of the final cohort comprising sixty-three AIS patients with M1-MCA occlusion, thirteen was the group of both poor collateral circulation and high relative PS and fifty belonged to the other. Thirty-three (52%) developed HT. ECASS II types were as follows: fifteen HI1(45%), eleven HI2(33%), four PH1(12%), three PH2(9%), and four SICH (12%). The mean time from symptom onset to HT was 5.21 ± 4.92 days. Interrater Reliability was excellent for collateral score (*k* = 0.95, *P* = 0.014) and relative PS (*k* = 0.91, *P* = 0.031).

Clinical characteristics were comparable between the patients with both poor collateral circulation and high relative PS (*n* = 13) and the other ones (*n* = 50) (Table 1). In the univariate logistic regression analysis, age, admission NIHSS, the new parameter—both collateral circulation and relative PS were all associated with HT. In the multivariate logistic regression analysis with three selected variables, only the new parameter—both collateral circulation and relative PS (odds ratio, 16.59; 95% confidence interval, 13.09–19.10; *P* < 0.001) was independent predictor of HT (Table 2). The area under the curve was 0.85 (95% confidence interval, 0.81–0.89). The sensitivity was 57%, specificity 97% and positive predictive value 92%, negative predictive value 58%.

**TABLE 1:** Clinical characteristics of patients.

	**The patients with both poor collateral circulation and high relative PS (*n* = 13)**	**The other patients (*n* = 50)**	***P* value**
Male, *n* (%)	8 (62)	24 (48)	0.744
Age, years	62.12 ± 8.71	63.23 ± 10.82	0.939
Admission NIHSS	17.22 ± 3.54	15.43 ± 4.81	0.331
Hypertension, *n* (%)	8 (62)	29 (58)	0.678
Hyperlipidemia, *n* (%)	3 (23)	8 (16)%	0.539
Diabetes mellitus, *n* (%)	11 (87)	30 (60)	0.729
Atrial fibrillation, *n* (%)	1 (8)	1 (2)	/
Previous stroke, *n* (%)	1 (8)	4 (8)	/
Current smoking, *n* (%)	5 (38)	22 (44)	0.723
Time to recanalization therapy, hours	4.42 ± 0.81	4.61 ± 0.65	0.396
Recanalization therapy			
Intravenous thrombolysis, *n* (%)	4 (31)	16 (32)	0.788
Endovascular thrombectomy, *n* (%)	3 (23)	10 (20)	0.532
Time to CTA/CTP, hours	2.43 ± 0.31	2.64 ± 0.13	0.623
Time to SWI, weeks	2.22 ± 0.57	2.53 ± 0.46	0.815
HT, *n* (%)	12 (92)	21 (42)	<0.001^∗^

*PS, permeability surface; NIHSS, National Institutes of Health Stroke Scale; CTA, CT angiography; CTP, CT perfusion; SWI, susceptibility-weighted imaging; HT, hemorrhagic transformation.*

Values were given as mean ± standard deviation or number of patients (%).

*P < 0.05.

**TABLE 2:** Univariate and multivariate logistic regression analysis for HT.

	**Univariate model**		**Multivariate model**	
	**Odds ratio (CI)**	***P* value**	**Odds ratio (CI)**	***P* value**
Age	1.23 (1.09–1.38)	0.037*	1.22 (1.05–1.39)	0.256
Admission NIHSS	1.32 (1.13–1.50)	0.042*	1.31 (1.12–1.51)	0.147
the new parameter—both collateral circulation and relative PS	16.57 (13.10–19.05)	< 0.001*	16.59 (13.09–19.10)	< 0.001*

HT, hemorrhagic transformation; CI, 95% confidence interval; NIHSS, National Institutes of Health Stroke Scale; PS, permeability surface.

*P < 0.05.

## Discussion

Our results indicated that the new integrated parameter—both collateral circulation and relative PS was strongly associated with HT after AIS. This study highlighted the promise of using multimodal CT parameter to predict HT in AIS patients with MCA-M1 occlusion.

Although purely clinical factors are useful in the decision-making process before rt-PA administration, in practice, most of these are insufficiently powerful in isolation to alter decision-making regarding treatment with thrombolysis, apart from absolute contraindications. This has led to the recent development of various HT prediction scoring systems using a variety of clinical and imaging-based predictors to estimate the pretreatment risk of HT (Hom et al., 2011; Bivard et al., 2016; Marsh et al., 2016; Wang et al., 2016; Kalinin et al., 2017; Li Q. et al., 2017; Li Y. et al., 2017; Liu et al., 2017; Puig et al., 2017). MRI is not routinely available in the acute setting, whereas multimodal CT is widely and rapidly accessible in most stroke centers.

Hemorrhagic transformation is a dynamic and complex phenomenon, and its pathophysiology is still not clear. The underlying mechanism may relate to impairment of the autoregulatory capacity of the cerebral vasculature and disruption of the blood-brain barrier (BBB) (Hom et al., 2011; Wang et al., 2016; Li Q. et al., 2017; Li Y. et al., 2017; Puig et al., 2017). Our study showed the poor collateral circulation and at the same time the high relative PS provided a key link to a higher incidence of HT. To our knowledge, this integrated parameter—both collateral circulation and relative PS has not been reported previously. Our results supported the view that the extent of hemodynamic derangement can help identify those at highest risk for HT and HT was the result of severe ischemic damage to the vessel walls that leads to BBB disruption (Hom et al., 2011; Wang et al., 2016; Li Q. et al., 2017; Li Y. et al., 2017; Puig et al., 2017). This new integrated parameter showed 97% specificity and 92% positive predictive value in predicting HT of AIS patients with MCA-M1 occlusion. For AIS patients with MCAO, these with poor collateral circulation on CTA and at the same time with high relative PS on CTP were at high risk for HT. It is the strength of our study. This may help clinicians to tighten current rt-PA treatment indications to exclude patients who may previously have been included.

CT angiography maximum intensity projection reconstruction is effective to evaluate leptomeningeal collateral flow. After MCA-M1 occlusion, leptomeningeal collateral is rapidly recruited to supply the ischemic area (Li Q. et al., 2017). PS map on CTP is intended to reflect the degree of BBB disruption, although it is potentially fraught with substantial variability due to differences in acquisition and postprocessing techniques, including the size and molecular charge of the contrast agent used. To a certain extent, our findings prompted that collateral blood flow may be a potential therapeutic target for reducing the incidence of HT. However, there was one uncertainty that should be taken into consideration that our results could not confirm that poor collateral circulation contributed to the increased PS or good collateral circulation contributed to the decreased PS for these two parameters were simultaneously acquired in this study.

This study has many limitations. First, this is a single-center study with a small sample size, that is, an insufficient number of patients with HT to stratify into symptomatic versus asymptomatic groups, or PH versus HI groups. The parameters threshold may not be accurate. Second, this study has the limitations and potential biases inherent in a retrospective study. The duration of the CTP acquisition is relatively short (50 s), which may make it questionable if it is truly measuring BBB permeability because our study is retrospectively conducted with imaging performed for clinical purposes rather than imaging optimized for research purposes. Therefore, further prospective research is required.

## Conclusion

In conclusion, the new integrated parameter—both collateral circulation and relative PS predicts HT in AIS patients with MCAO. The incorporation of readily available multimodal CT parameter into the clinical decision-making algorithm may allow for a greater appreciation of the risk-benefit profile before decisions about thrombolytic therapy in AIS. Further analysis with a larger number of patients and clinical end points in an independent data set is required to optimize and validate the methodology and potentially make this multimodal image protocol more applicable in the clinical setting.

## Data Availability Statement

The raw data supporting the conclusions of this article will be made available by the authors, without undue reservation.

## Ethics Statement

The studies involving human participants were reviewed and approved by the Ethics Committee of Huashan Hospital, Fudan University. Written informed consent for participation was not required for this study in accordance with the national legislation and the institutional requirements.

## Author Contributions

CL, ZY, XF, and YY were responsible for study concept and the design. CL, XH, LL, CS, and HY collected the data. CL, XH, LL, and CS analyzed the data and conducted the statistical analysis. CL wrote the manuscript. ZY, XF, and YY supervised the manuscript and provided technical or information support. All authors approved the final version of the manuscript.

## Conflict of Interest

The authors declare that the research was conducted in the absence of any commercial or financial relationships that could be construed as a potential conflict of interest.

## Publisher’s Note

All claims expressed in this article are solely those of the authors and do not necessarily represent those of their affiliated organizations, or those of the publisher, the editors and the reviewers. Any product that may be evaluated in this article, or claim that may be made by its manufacturer, is not guaranteed or endorsed by the publisher.

## Abbreviations

AIS: acute ischemic stroke
BBB: blood-brain barrier
CTA: CT angiography
CTP: CT perfusion
HI: hemorrhagic infarction
HT: hemorrhagic transformation
MCA: middle cerebral artery
MCAO: middle cerebral artery occlusion
MRI: magnetic resonance imaging
NCCT: no-contrast CT
NIHSS: National Institutes of Health Stroke Scale
PH: parenchymal hematoma
PS: permeability surface
ROI: regions of interest
rt-PA: recombinant tissue plasminogen activator
SICH: symptomatic intra cerebral hemorrhage
SWI: susceptibility-weighted imaging.
