# Supplementary material for: Prognostic Value of a New Integrated Parameter—Both Collateral Circulation and Permeability Surface—in Hemorrhagic Transformation of Middle Cerebral Artery Occlusion Acute Ischemic Stroke: Retrospective Cohort Study
Source: Front Aging Neurosci. 2021 Aug 25;13:703734. doi: 10.3389/fnagi.2021.703734 (PMC8424095; doi:10.3389/fnagi.2021.703734)
Supplement: Supplementary file 1 [file Data_Sheet_1.docx]

**Supplementary Figure I.** Study flow chart

Abbreviations: MCAO = middle cerebral artery occlusion; CTA = CT angiography; CTP = CT perfusion; SWI = susceptibility-weighted imaging.

**Supplementary Table I.** Comparison of baseline characteristics between patients involved and not involved in this study.

|  | MCAO AIS involved  in study (n=63) | MCAO AIS not involved  in study (n=118) | *P* value |
| --- | --- | --- | --- |
| Male, n (%) | 32(51) | 58(49) | 0.223 |
| Age, years | 63.81±9.72 | 65.13±11.21 | 0.312 |
| Admission NIHSS | 16.12±4.91 | 14.66±6.27 | 0.686 |
| Hypertension, n (%) | 37(59) | 71(60) | 0.931 |
| Hyperlipemia, n (%) | 11(17) | 19(16) | 0.726 |
| Diabetes mellitus, n (%) | 41(65) | 83(70) | 0.137 |
| Atrial fibrillation, n (%) | 2(3) | 5(4) | 0.512 |
| Previous stroke, n (%) | 5(8) | 11(9) | 0.655 |
| Current smoking, n (%) | 27(43) | 59(50) | 0.204 |

Abbreviations: MCAO = middle cerebral artery occlusion; AIS = acute ischemic stroke; NIHSS = National Institutes of Health Stroke Scale.

Values are given as mean ± standard deviation or number of patients (%).
